# Performance of Bleach Method Sputum Smear Microscopy for the Diagnosis of Tuberculosis in a Highly Endemic District in Lima, Peru

**DOI:** 10.3390/ijerph20010135

**Published:** 2022-12-22

**Authors:** Jaime Rosales-Rimache, Magda Nunayalle-Vargas, Lenin Rueda-Torres, Jorge Inolopú-Cucche

**Affiliations:** 1 Facultad de Medicina, Universidad Privada Norbert Wiener, Lima 39, Peru; 2 Laboratorio Clínico, Hospital III Suárez Angamos, Essalud, Lima 18, Peru; 3 Facultad de Ciencias y Filosofía, Universidad Peruana Cayetano Heredia, Lima 31, Peru; 4 Facultad de Administración y Salud Pública, Universidad Peruana Cayetano Heredia, Lima 31, Peru

**Keywords:** tuberculosis, bacilloscopy, sputum

## Abstract

Background: Sputum smear microscopy (SSM) is a screening test used to diagnose tuberculosis (TB); however, its performance and sensitivity are relatively low, which can lead to false negatives. We designed a cross-sectional study to estimate the performance of SSM that includes a pretreatment based on sputum digestion with bleach (sodium hypochlorite) for the diagnosis of TB. Methods: We evaluated 73 sputum samples from patients with a diagnosis of TB confirmed by the Xpert MTB/RIF test and 114 samples from patients without TB. We performed sputum digestion using a 5% sodium hypochlorite solution, centrifuged at 2000 rpm for 15 min. We prepared smears for direct and bleach-treated SSM and used Ziehl–Neelsen staining. Results: The bleach-treated SSM obtained absolute identification of the cases of TB confirmed by the Xpert test, compared to 95.9% identified by the direct smear method (without bleach treatment). We also found a significant increase (*p* < 0.001) in the recovery of acid-fast bacilli (AFB) obtained by the bleach-treated SSM (293.8 ± 215.1 AFB) compared to the direct SSM method (222.9 ± 195.5 AFB). The AUC of the bleach-treated SSM and direct SSM was 100% and 96.6%, respectively. Conclusion: The bleach-treated SSM performs better than the direct SSM in identifying AFB and increasing the bacillary count in the sputum samples.

## 1. Introduction

Tuberculosis (TB) is a disease that causes approximately 10 million infections and more than 1.5 million deaths worldwide each year, and it is considered the leading infectious cause of death in the world [1]. Among its priorities, the World Health Organization considers the eradication of TB as a millennium development goal, especially in endemic countries [2]. In this regard, Peru has the second-highest TB burden in Latin America and the Caribbean [3], and it is one of the 30 countries with the highest burden of multidrug-resistant TB in the world [4]. However, despite the efforts of the Tuberculosis Prevention and Control Directorate of the Ministry of Health of Peru [5], TB remains one of the five leading causes of death in this country. Although its incidence in the years just prior to COVID-19 had decreased, its rates are is still very high [6]. Therefore, the diagnosis of TB is essential to ensure early treatment, since this disease can be cured in its early stages [7]. In this sense, the use of tools and instruments for the identification of the causal agent, *Mycobacterium tuberculosis*, is a method that must improve within health establishments.

One of the most critical techniques in screening for TB is the study of sputum smear microscopy (SSM). This consists of evaluating the sputum sample, which allows for the preparation of smears on slides and subsequent Ziehl–Nielsen staining to show acid-fast bacilli (AFB), which are characteristic of *Mycobacterium tuberculosis* [8]. However, SSM has limitations that reduce diagnostic performance and sensitivity, especially in cases where the bacillary load is low [9]. Preanalytical factors involved in the quantity and quality of the sputum sample obtained can potentially increase the rate of false negatives [10]. This highlights the presence of interferers or contaminants in the staining and reading of slides, as well as the limitations in obtaining serial samples on the same or different days, since sometimes, patients do not return [11].

In recent years, various modifications to the SSM have been sought, ranging from pretreatment processes on the sputum sample [12] to replacements in the staining used to identify *Mycobacterium tuberculosis* [13], all to increase the performance and sensitivity of the test. One of these modifications is based on the treatment with sodium hypochlorite (NaOCl) for the liquefaction and concentration of the sputum samples through an easy-to-implement and low-cost process known as the bleach method. Many investigations have explored this method, with results supporting its use as a mucolytic agent that improves the visibility of AFB, allowing for an increased sensitivity of SSM [14,15,16]. However, it is necessary to obtain evidence regarding the performance of the bleach method in different contexts. In this sense, our research aimed to assess the performance of SSM using a sputum digestion technique with sodium hypochlorite in diagnosing TB, compared with the conventional method based on direct SSM (without treatment).

## 2. Materials and Methods

### 2.1. Study Area and Design

We designed a cross-sectional study to evaluate 187 participants for the diagnosis of TB in December 2018 at the Ollantay Maternal and Child Center, a primary care health center located in the south of Lima, the capital of Peru. This health center belongs to the Pamplona Alta sector, District of San Juan de Miraflores, one of the ten districts with the highest prevalence of TB in Lima [17], and it cares for patients of all ages with suspected TB. These conditions support the development of the study in this context.

### 2.2. Participants

We calculated the sample size (*n* = 187 patients) considering expected sensitivity and specificity values of 95 and 92%, and a precision and confidence level of 5 and 95%. We selected participants from the passive surveillance carried out by the health center in people with symptoms suggestive of TB. We included sputum samples from 73 patients diagnosed with pulmonary TB (cough with expectoration for more than two weeks) and defined according to the Xpert MTB/RIF test, considered as our reference test. Likewise, we included sputum samples from 114 patients of the health facility without TB, as confirmed by the Xpert MTB/RIF test.

### 2.3. Techniques and Procedures

#### 2.3.1. Sputum Samples Collection

We collected two consecutive early morning sputum samples per participant, obtained by spontaneous coughing effort or induced by inhalation of physiological saline. Sputum samples in sufficient volumes (5–10 mL) were kept at room temperature for 2 h before processing; otherwise, they were held at 2–8 °C for 24 h until processing. Poorly collected sputum samples (saliva, for example) or those that did not meet the established conservation parameters were excluded. We carried out the procedures for collecting sputum samples following the training manual from the Ministry of Health of Peru for managing TB [18].

#### 2.3.2. Sputum Samples Treatment

The first sputum sample was used to perform the direct SSM (without bleach treatment) and to determine the presence of *Mycobacterium tuberculosis* complex DNA by hte Xpert MTB/RIF test (Cepheid, Sunnyvale, CA, USA) within seven days of sample collection, according to the manufacturer’s recommendations. The second sputum sample was bleach treated according to the procedure described by Ongkhammy et al., which obtained satisfactory results, increasing the sensitivity of the diagnosis of pulmonary TB [19]. This procedure consists of adding 10 mL of 5% sodium hypochlorite and mixing by stirring for 15 min at room temperature. Then, 2–7 mL of the homogenate was transferred to a 15 mL conical tube, and an equal volume of distilled water was added and centrifuged at 2000 rpm for 15 min. We removed a volume of sediment and deposited a drop on a slide, allowing it to dry at room temperature.

#### 2.3.3. Sputum Smears Staining and Microscopy

The sputum smear samples (direct and bleach-treated) were fixed to the slide by flame heat from a Bunsen burner. Subsequently, we proceeded with the primary staining with carbol fuchsin for 5 min, which was gently exposed to the flame heat until it achieved vapor emission, repeating the process up to 3 times. Excess fuchsin was removed by washing with tap water and acid alcohol (3 mL 99.9% chloridric acid and 97 mL 95% ethanol) for 2 min until a pale pink color was obtained, and then the sample was washed again with tap water and stained with methylene blue for 30–60 s. The SSM process followed the recommendations of the training manual for the management of TB from the Ministry of Health of Peru [18]. Finally, the slides were washed with tap water and dried at room temperature.

The sputum smears were evaluated with light-emitting diode (LED) microscopy at a magnification of 100×. We aimed to identify AFB, characterized by a bright red color on a blue background, with rod-shaped and straight, slightly curved, small (between 1–4 µm) structures arranged in groups of 3–10 AFB, according to the procedures validated by the National Institute of Health of Peru [20].

Microscopists are trained in the reading of SSM and are constantly evaluated with proficiency tests as part of an external quality control program established by the National Institute of Health of Peru [21]. As part of the study, the microscopists were blinded in recognizing what type of SSM they evaluated (direct or bleach-treated).

### 2.4. Statistical Analysis

We described the general characteristics of the participants. We evaluated the performance of the bleach-treated SSM using parametric ROC (receiver operating characteristic) analysis and the calculation of the area under the curve (AUC), comparing the results against the Xpert MTB/RIF test results. Likewise, we estimated the standard error and its 95% confidence interval. The comparison of the results between the direct and bleach-treated SSM and Xpert MTB/RIF test was evaluated by the Chi-square test, considering a *p*-value less than 0.05 as a significant difference. On the other hand, we assessed the bacillary counts between the direct and bleach-treated SSM using the non-parametric Wilcoxon test, considering a *p*-value less than 0.05 as a significant difference. We performed statistical analysis using Stata v.17 software (StataCorp, College Station, TX, USA).

### 2.5. Ethical Aspects

The study was reviewed and approved on 19 February 2019, by the South Lima Regional Directorate of the Ministry of Health of Peru, with Official Letter No. 114–2019-CMI Ollantay-DLS. The participants gave their informed consent to use their sputum samples in the investigation, after a prior process informing the patient of the benefits and risks involved in the study. The biological material was coded and handled with total discretion and confidentiality, respecting every bioethical principle used in human research.

## 3. Results

As part of a case finding, we collected 374 sputum samples from 187 participants. No sputum samples were excluded based on study eligibility criteria. The participants were 75 males and 112 females, with a mean age of 43.3 ± 19.9 years, who were classified into two groups: 114 MTB-negative and 73 MTB-positive, according to the Xpert MTB/RIF test. Of the group of participants who were MTB-positive, the mean ages were 47.6 ± 19.1, and 57.5% were men, while in the group who were MTB-negative, the mean ages were 36.7 ± 19.4 years, and 71.1% were women.

Table 1 shows the semiquantitative results of the reading by microscopy of the direct and bleach-treated SSM using crosses. Of 187 sputum samples, the direct SSM detected the presence of AFB in 70 (37.4%) samples, while we detected the presence of AFB in 73 (39.0%) samples using the bleach-treated SSM (*p* = 0.080, McNemar test). We observed that the bleach-treated SSM increased the percentage of AFB. Moreover, it increased the bacillary count in the paucibacillary results by one to two grades when compared to the results obtained with the direct SSM. Moreover, the Cohen’s kappa coefficient between the direct and bleach-treated SSM was 0.301 (IC95: 0.187–0.376).

Table 2 compares the direct and bleach-treated SSM results with the Xpert MTB/RIF test results. In the MTB-positive group, the bleach method identified the presence of AFB in all sputum samples, whereas the direct SSM assessed AFB-positive sputum samples in only 70 (95.9%) of the sputum samples. On the other hand, both direct and bleach-treated SSM showed negative results in all samples confirmed as MTB-negative by the Xpert-MTB/RIF test. Thus, the direct SSM had a sensitivity and specificity of 95.9% (CI95: 88.5–99.1%) and 100% (CI95: 96.8–100.0%), respectively, with a Cohen’s kappa coefficient of 0.966, relative to the Xpert MTB/RIF test. The bleach-treated SSM exhibited a sensitivity and specificity of 100.0% (CI95.1–100.0%) and 100.0% (IC95: 96.8–100.0%), respectively, compared to the results of the Xpert MTB/RIF test. The sensitivity of the bleach-treated SSM and the direct method exhibited significant differences (*p* < 0.01).

**Figure A1:**
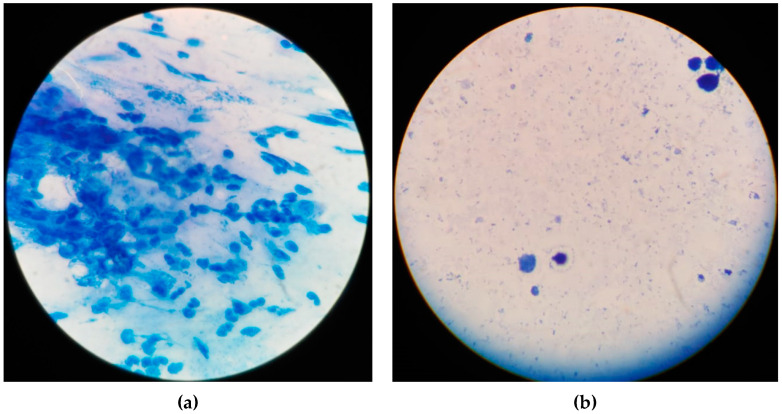
Negative smear microscopy for sputum sample 4481. (**a**) Directed SSM (**b**) The bleach SSM. Magnification: 100× in immersion.

**Figure A2:**
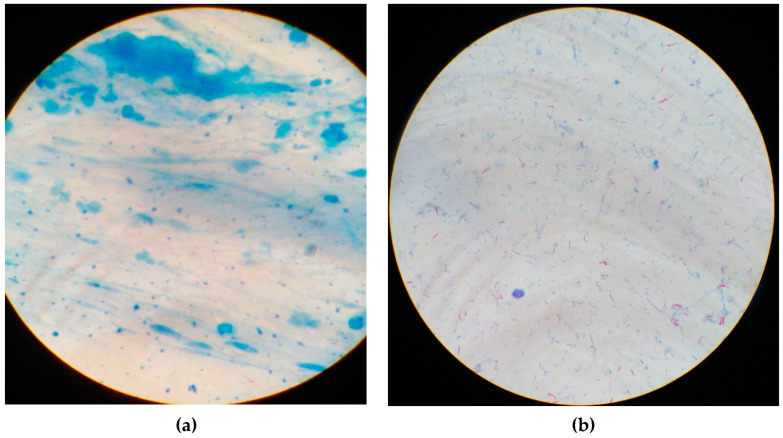
Negative smear microscopy for sputum sample 4475. (**a**) Directed SSM: 297 AFB, 2+ (**b**) The bleach SSM: 401 AFB, 3+. Magnification: 100× in immersion.

**Figure A3:**
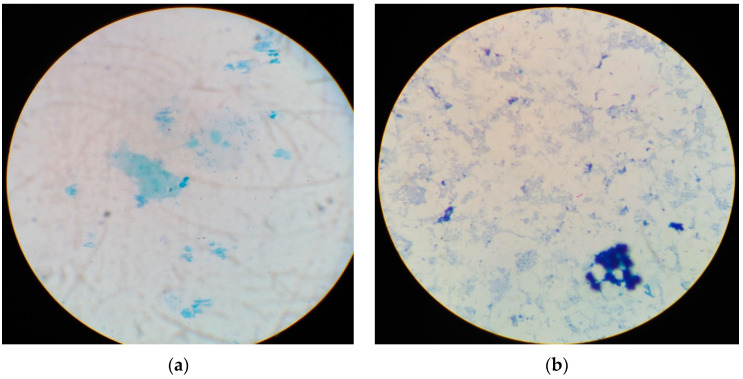
Negative smear microscopy for sputum sample 4252. (**a**) Directed SSM: 2 AFB (**b**) The bleach SSM: 80 AFB, 1+. Magnification: 100× in immersion.

**Figure A4:**
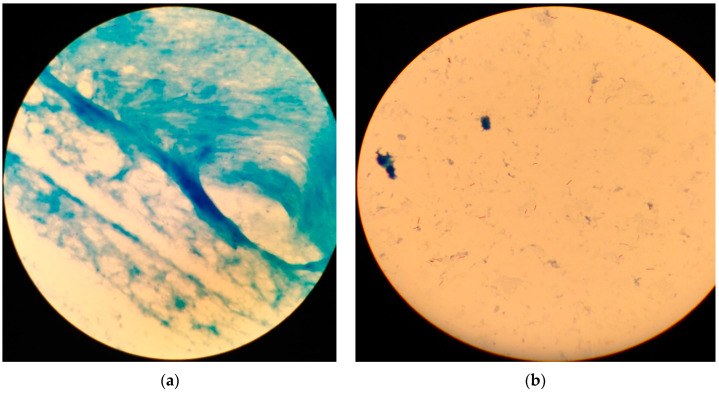
Negative smear microscopy for sputum sample 141. (**a**) Directed SSM: 312 AFB, 3+ (**b**) The bleach SSM: 541 AFB, 3+. Magnification: 100× in immersion.

Table 3 shows the distribution of the median bacillary count obtained from the direct and bleach-treated SSM, according to categories of sex and age. Regarding sex, the median of the bacillary count was significantly higher with bleach-treated SSM than with direct SSM (*p* < 0.01). In men, the median bacillary count obtained by direct and bleach-treated SSM were 213 (IQR: 29–301 AFB) and 286 (IQR: 92–381 AFB), respectively. In women, the median bacillary count obtained by direct and bleach-treated SSM were 119 (IQR: 92–367 AFB) and 336 (IQR: 142–453 AFB), respectively. According to age groups, significant differences were found in patients between 18 and 40 years old, while in those older than 40 years, we found no differences. In general, the median bacillary count in all samples was significantly higher with the bleach method (213 (IQR: 71–312 AFB)) than with the direct SSM method (286 (IQR: 125–392 AFB)) (see Appendix A: Figure A1, Figure A2, Figure A3 and Figure A4). Therefore, there is an increase in the median of 34.4% regarding the total bacillary count using the bleach method compared to the direct method.

According to the microscopists in the study, the slides corresponding to the bleach method present a lower amount of cell debris and contaminants that interfere with the SSM (Appendix A). Regarding the performance of the direct and bleach-treated SSM, Table 4 show the value of the area under the curve. In the case of the direct SSM, we obtained a value of 98.0%, lower than that obtained by the bleach method (100%). However, no significant difference between direct and bleach-treated SSM was obtained (*p* = 0.079).

## 4. Discussion

The results of bleach-treated SSM using the digestion process with sodium hypochlorite showed a higher performance than did the direct SSM, although without statistically significant differences. However, the increase in the AFB count by the bleach-treated SSM was significant compared to the direct SSM, obtaining a recovery gain in the count of 34.3%. The AFB recovery in the sputum samples was lower than that reported by other researchers who have used other concentration systems, such as Aerospray^®^ TB Slide Stainer (ELITechGroup, Puteaux, France), showing a recovery of 49.5% compared to that of the direct SSM [22]. However, these systems were more complicated to implement. Our results are significant because the bleached method is simple, safe, and cheap, and it improves the performance of SSM and the sensitivity of TB diagnosis.

A large amount of evidence reports an increase in AFB gradation or AFB count after treatment with 5% bleach and centrifugation, which supports its use, especially in low-resource contexts [23]. However, contrary to our results, Chew et al. [24] showed a significant reduction in the AFB count obtained from bleach-treated SSM compared to that obtained from direct SSM. The bleach-treated procedure used by Chew et al. is similar to ours, but it included spontaneous sedimentation for 16 h, which could have affected the integrity of *Mycobacterium tuberculosis*.

The studies comparing direct SSM with the gold standard methods, such as culturing or the Xpert MTB/RIF test, have shown low sensitivity [25]. In our case, of all the MTB-positive samples evaluated by the Xpert-MTB/RIF test, the direct SSM reached 95.9% positivity, while the bleach-treated SSM obtained 100% agreement. Other studies have shown alternative ways to identify AFB, for example, by fluorescence microscopy, with findings similar to those reported in our study. Mizuno K. et al. showed a 98.2% concordance between fluorescent and conventional SSM [26]; on the other hand, other authors have reported an accuracy of up to 100% for SSM in cytological smears using simple Ziehl–Neelsen staining [27], a parameter that we did not evaluate in this research.

The results of the bleach method have generated a sensitivity of 100% (positive in those who have MTB-positive samples evaluated by the Xpert-MTB/RIF test), a value higher than that reported by Farnia et al. They found a sensitivity of 83%, 78%, and 80% in sputum samples treated with N-acetyl cysteine, sodium hypochlorite, and chitin, respectively, compared with the results of culturing [28]. The bleach method provides a fast, safe, and economical method compared to methods using sodium hydroxide and centrifugation [29]. Other treatments with ammonium sulfate or chlorine are subjected to sedimentation [30], but with the disadvantage of using chemical inputs requiring greater safety conditions for handling.

Microscopic identification of AFB is consistently improved with the use of high-quality light. In our case, we guarantee clear reading by using an LED system, generating clear images with good resolution. Although many investigations recommend using fluorescence microscopy to improve reading sensitivity, this procedure is expensive and challenging in the primary care laboratories of low- and middle-income countries [31]. The efficacy of LED microscopy has been evaluated against fluorescence, and researchers recommend it as an alternative to conventional light microscopy [32].

The bleach method increases the bacillary count, improves the sensitivity of SSM, reduces the presence of cellular debris, and improves the microscopic background. The digestion capacity of the bleach method has been reported as efficient, especially in samples of a mucoid rather than a purulent nature [33], which could help to significantly reduce read times in SSM [26]. A systematic review indicates that the use of the bleach method offers fewer benefits than expected, although this is due to the studies that did not meet the GRADE quality criteria [34]. Therefore, more exploration of the usefulness of the bleach method is essential.

The study’s limitations may refer to using the Xpert MTB/RIF test as a reference standard, considering that it involves a closed system (GeneXpert^®^ system) (Cepheid, Sunnyvale, CA, USA). In this regard, we could not to access microbiological culture results, since their results require a long time period; however, we did obtain results from the Xpert MTB/RIF test, which has a sensitivity and specificity very close to that of microbiological culture method [35,36]. In addition, the Xpert MTB/RIF test is part of the line of preliminary diagnostic tests that is being widely implemented in Peru. Despite this, we did not have access to the semiquantitative results of the participants with an MTB complex positive result (very low, low, medium, and high), which could help to better explain the AFB grading obtained by SSM [37]. Additionally, we did not obtain interobserver comparison results between the microscopists who performed the SSM. Moreover, in our study, the first sputum sample was used to carry out the direct method, and the second for the bleach method, which contrasts with similar studies that carry out these treatments from a single sample [19,29,38,39]. The collection of sputum at different times could affect the results, if there were significant differences in AFB counts between the first and second sputum samples. Finally, we do not know the HIV seropositivity status of the study participants, which could influence the presence of AFB in the sputum samples [40]. These aspects should be considered in subsequent studies in order to generate consistent results.

Our results highlight the importance of using a procedure that improves SSM findings in identifying, diagnosing, monitoring, predicting, and treating TB. As has been observed in the investigation, sodium hypochlorite is essential to guarantee the digestion, liquefaction, and clearance of the sputum samples. This chemical is easily accessible due to its high availability in primary care health centers, and concentrations similar to the one used in our study have shown sterilizing capacity, which could help prevent biological risks [24]. However, its use makes it unfeasible and unsuitable to proceed with tests such as microbiological culture. However, the alternative of processing SSM before the digestion process is relevant, especially in handling pediatric sputum samples, considering that their quality is poor, and it is essential to increase the probability of identifying AFB. Finally, our study has generated necessary evidence on the bleach-treated SSM, which is likely to reduce false negatives using a direct smear. The bleach method is an easy alternative for use in primary care laboratories in low and middle-income countries.

## 5. Conclusions

The performance of bleach-treated SSM in diagnosing TB was very high. Likewise, it significantly increases AFB recovery, which generates higher scores on the crossing scale. The digestion method can be introduced in routine laboratories at primary care establishments, since it does not require high-tech equipment and can be executed by health professionals, technicians, and microbiology personnel after only a brief training period. Moreover, highlighting the importance of digestion with sodium hypochlorite reduces the possibility of occupational infections during the processing of sputum sampling and smear microscopy studies in general. Finally, although the blanking method has been explored in many investigations, our results show that the procedure can be carried out easily, quickly, and efficiently, guaranteeing the validity required for a screening method for diagnosing TB.

## Figures and Tables

**Table 1:** Comparison between the bacillar count of direct and bleach-treated SSM.

Bleach-Treated SSM	Direct SSM					
	Neg	+/−	+	++	+++	Total
Neg	114 (97.4)	0	0	0	0	114
+/−	0	1	3	0	0	4
+	0	5	9	2	1	17
++	3	2	9	6	2	22
+++	0	0	2	8	20	30
Total	117	8	23	16	23	187

**Table 2:** Comparison between direct and bleach-treated sputum smear microscopy (SSM) with results from the Xpert MTB/RIF test.

SSM	Xpert MTB/RIF Test	
	MTB-Negative (*n* = 114)	MTB-Positive (*n* = 73)
Direct SSM		
Negative	114 (100.0%)	3 (4.1%)
Positive	0 (0.0%)	70 (95.9%)
Bleach-treated SSM		
Negative	114 (100.0%)	0 (0.0%)
Positive	0 (0.0%)	73 (100.0%)

**Table 3:** Comparison of bacillary count between direct and bleach-treated sputum smear microscopy (SSM).

Demographics	Median (Interquartile Range)		^a^*p*-Value
	Direct SSM	Bleach-Treated SSM	
Sex			
Male	213 (29–301)	286 (92–381)	<0.001
Female	119 (92–367)	336 (142–453)	<0.001
Age group			
<18 years	---	---	---
18–40 years	106 (26–312)	286 (125–401)	<0.001
>40 years	297 (182–317)	302 (96–367)	0.768
Total	213 (71–312)	286 (125–392)	<0.001

^a^ Wilcoxon’s nonparametric test.

**Table 4:** Performance of direct and bleach-treated sputum smear microscopy (SSM).

SSM	AUC	SE	CI95	*p*-Value ^a^
Direct	97.95%	1.2%	92.5–100.0%	0.079
Bleach method	100.0%	0.0%	100.0–100.0%	

AUC: Area under the curve; SE: standard error; CI95: confidence interval at 95%. ^a^ Obtained from the Chi-square test.

## Data Availability

The data presented in this study are available on request from the corresponding author. The data are not publicly available due to privacy considerations.
